# Supplementary material for: Sustained-input switches for transcription factors and microRNAs are central building blocks of eukaryotic gene circuits
Source: Genome Biol. 2013 Aug 23;14(8):R85. doi: 10.1186/gb-2013-14-8-r85 (PMC4054853; doi:10.1186/gb-2013-14-8-r85)
Supplement: Additional file 5 — HTML Browsable Motif Output. Zipped folder containing all WaRSwap and FANMOD motif output, viewable in a web browser. [file gb-2013-14-8-r85-S5.ZIP › HTML_browsable_motif_output/WaRSwap_ath_tair9/sigs_WSWRSLDraw_2-2000.pvals.heatmaps.html/motif_id_46_011101002_tftype_ath_upstream_-1000_0.html]

```
BG_MODEL = Rnew2
MOTIF_ID = 46_011101002
TF_TYPE = ath
UPSTREAM = -1000_0


PVals
FN_0.2	FN_0.4	FN_0.6	FN_0.8
dg_60.genes	0.059	0.005	0.267	0.422
dg_70.genes	0.058	0.006	0.254	0.409
dg_80.genes	0.059	0.005	0.271	0.438

ZScores
FN_0.2	FN_0.4	FN_0.6	FN_0.8
dg_60.genes	1.481	2.564	0.463	-0.514
dg_70.genes	1.474	2.611	0.476	-0.501
dg_80.genes	1.463	2.587	0.453	-0.516

StDevs
FN_0.2	FN_0.4	FN_0.6	FN_0.8
dg_60.genes	131873.445	44583.637	4144.824	250.837
dg_70.genes	128051.87	44011.202	4176.368	252.217
dg_80.genes	129564.965	43464.844	4198.596	236.601
```
